# The complete chloroplast genome sequence of *Phalaenopsis lowii* (Orchidaceae)

**DOI:** 10.1080/23802359.2019.1674715

**Published:** 2019-10-15

**Authors:** Jie-Yu Wang, Zhong-Jian Liu, Guo-Qiang Zhang, Chang-Cao Peng

**Affiliations:** a State Key Laboratory for Conservation and Utilization of Subtropical Agro-bioresources, South China Agricultural University, Guangzhou, China;; b Guangdong Key Laboratory for Innovative Development and Utilization of Forest Plant Germplasm, College of Forestry and Landscape Architecture, South China Agricultural University, Guangzhou, China;; c Shenzhen Key Laboratory for Orchid Conservation and Utilization, The National Orchid Conservation Centre of China and The Orchid Conservation and Research Centre of Shenzhen, Shenzhen, China;; d Key Laboratory of National Forestry and Grassland Administration for Orchid Conservation and Utilization at College of Landscape Architecture, Fujian Agriculture and Forestry University, Fuzhou, China

**Keywords:** Moth orchid, subgenus Parishianae, *Phalaenopsis lowii*, plastid genome

## Abstract

In this paper, we obtained and characterized the complete chloroplast genome sequence of a unique moth orchid, *Phalaenopsis lowii*. The total plastid genome size is 146,834 bp, containing a large single copy (LSC) region (84,469 bp) and a small single-copy region (10,477 bp) that were separated by two inverted repeats (IRs) regions (25,944 bp). We annotated 110 unique genes, within which there are 76 protein-coding genes, 30 tRNA genes, and 4 rRNA genes. Phylogenetic analysis indicated the *P. lowii* showed a sister relationship with subgenus *Phalaenopsis* clade.

*Phalaenopsis* (Vandeae, Orchidaceae) is a remarkable genus with highly ornamental value, and its showy flower attracts millions of people (Van 2018). So far, *Phalaenopsis* is divided into four subgenera, subgen. *Phalaenopsis*, subgen. *Parishianae*, subgen. *Hygrouchilus* and subgen. *Ornithochilus* (Kocyan and Schuiteman 2014; Li et al. 2014, 2016). Although there were three complete chloroplast genome of *Phalaenopsis* have been published (Chang et al. 2006; Jheng et al. 2012; Kim et al. 2016), these sequenced species are all from subgen. *Phalaenopsis* while the chloroplast genome of subgen. *Parishianae* is still lack. Species belonging to subgen. *Parishianae* are also well-known breeding parent, and their genetic data are required to improve our understanding on these species and also promote the breeding of moth orchid. So, we choose *P. lowii*, a member of subgen. *Parishianae* for a complete chloroplast genome.

*Phalaenopsis lowii* was sampled from National Orchid Conservation Centre in Guangdong province of China (114°19'01''E, 22°60'34'' N). A voucher specimen (*noccphal004*) was deposited in the Herbarium of National Orchid Conservation Centre, Shenzhen, China. The total DNA was extracted from the young leaves of the voucher specimen of *Phalaenopsis lowii* and the genome was sequenced using Illumina HiSeq 2000 platform (Illumina, San Diego, CA). Then the raw reads were mapped into the three published *Phalaenopsis* chloroplast genomes to obtain the plastid source reads. Then we used PLATANUS to assemble the reads into complete genome with the artificial modification. Annotation of the chloroplast genome was conducted by online pipeline Geseq (https://chlorobox.mpimp-golm.mpg.de/geseq.html) and Geneious 2019.0.3, and we confirmed the IR boundaries for the draft chloroplast genome by BLAST. The complete chloroplast genome of *P. lowii* was submitted to GenBank with the accession number of MN385684.

The total chloroplast genome size of *P. lowii* is 146,834 bp, and is slightly less than species belonging to subgenus *Phalaenopsis*, such as *P. aphrodite* subsp. *formosa* (148,964 bp), *P. equestris* (148,959 bp) and *P. ‘*Tiny Star’ (148,918 bp). It contains a large single copy (LSC) region (84,469 bp) and a small single-copy region (10,477 bp), which were separated by two inverted repeat (IRs) regions (25,944 bp). The overall GC contents of the plastid genome were 36.9%. In total, 110 unique genes were annotated, including 76 protein-coding genes, 4 rRNA genes, and 30 tRNA genes. The protein-coding genes, rRNA genes, and tRNA genes account for 69.0, 3.6, and 27.3% of all annotated genes, respectively. All *ndh* genes for *P. lowii* are non-functional, and the *ndhA*, *ndhF*, and *ndhH* genes were completely absent as previous description by Chang et al. (2006).

The maximum-likelihood phylogenetic tree was generated using RAxML (Stamatakis 2014) based on the complete chloroplast genome of *P. lowii*, three published moth orchids, and also the other eleven orchids. *Cattleya crispate* and *Masdevallia coccinea* were used as an outgroup based on their phylogenetic position as Givnish et al. (2015). The phylogenetic tree showed that *P. lowii* was sister to other three *Phalaenopsis*, and those formed a single clade (Figure 1). The topology is similar to Givnish et al. (2015), where the Vandeae displayed a sister relationship to Cymbidieae. This published *L. lowii* chloroplast genome will provide useful information for phylogenetic studies and future breeding in *Phalaenopsis* even in Orchidaceae.

**Figure 1.**
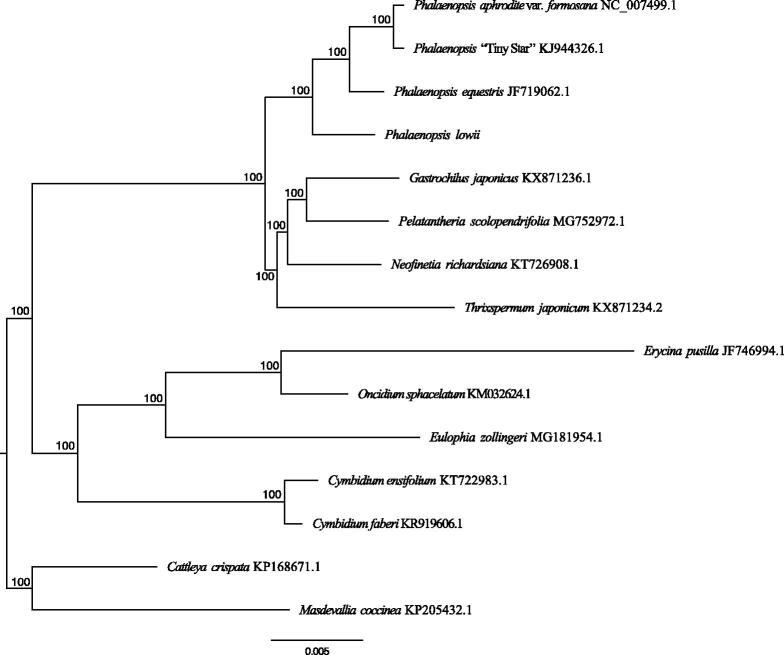
Maximum-likelihood phylogenetic tree reconstructed by RAxML based on complete chloroplast genome sequences from *P. lowii*, three *Phalaenopsis*, and 11 other orchids with species from Epidendreae as outgroup (*Cattleya crispate* and *Masdevallia coccinea*). Numbers on branches are bootstrap support values.
